# Elevated Expression of TLR2 in Aging Hearts Exacerbates Cardiac Inflammatory Response and Adverse Remodeling Following Ischemia and Reperfusion Injury

**DOI:** 10.3389/fimmu.2022.891570

**Published:** 2022-04-14

**Authors:** Yufeng Zhai, Lihua Ao, Qingzhou Yao, Erlinda The, David A. Fullerton, Xianzhong Meng

**Affiliations:** Department of Surgery, University of Colorado Denver, Aurora, CO, United States

**Keywords:** aging, TLR2, inflammation, myocardial ischemia, remodeling

## Abstract

This study tested the hypothesis that Toll-like receptor 2 (TLR2) augments the inflammatory responses and adverse remodeling in aging hearts to exacerbate myocardial injury and cardiac dysfunction.

**Methods:**

Old (20-22 months old) and adult (4-6 months old) mice of C57BL/6 wild-type and TLR2 knockout (KO) were subjected to coronary artery ligation (30 minutes) and reperfusion (3 or 14 days). Left ventricle function was assessed using a pressure-volume microcatheter. Cardiac infarct size was determined by histology. Levels of vascular cell adhesion molecule-1 (VCAM-1), intercellular adhesion molecule-1 (ICAM-1), matrix metalloproteinase 9 (MMP 9), and collagen I in non-ischemic myocardium were assessed by immunoblotting. Monocyte chemoattractant protein-1 (MCP-1), keratinocyte chemoattractant (KC), and interleukin-6 (IL-6) levels in ischemic and non-ischemic myocardium were measured by enzyme-linked immunosorbent assay (ELISA). TLR2 expression in the myocardium of untreated wild type mice was also measured by immunoblotting.

**Results:**

Higher levels of MCP-1, KC, IL-6 were induced in both ischemic and non-ischemic myocardium of old wild type mice at day 3 and 14 following ischemia/reperfusion (I/R) than those of adult wild type mice. The hyper-inflammatory responses to I/R in aging hearts were associated with elevated levels of myocardial TLR2. TLR2 KO markedly down-regulated the expression of MCP-1, KC, IL-6, ICAM-1 and VCAM-1 in aging hearts at day 3 and 14 following I/R. The down-regulated inflammatory activity in aging TLR2 KO hearts was associated with attenuated production of MMP 9 and collagen I at day 14 and resulted in reduced infarct size and improved cardiac function.

**Conclusion:**

Elevated expression of myocardial TLR2 contributes to the mechanism by which aging exacerbates the inflammatory responses, adverse remodeling and cardiac dysfunction following myocardial I/R in aging.

## Introduction

Advanced age is a major risk factor for cardiovascular disease. Ischemic heart disease is a primary cause of morbidity and mortality in old patients (1). Indeed, old people have a greater likelihood of cardiac dysfunction following myocardial ischemia and reperfusion (I/R), and are more likely to develop other complications, including left ventricle free wall rupture and acquired ventricular septal defect (2, 3). It is well known that myocardial I/R injury induces the over production of inflammatory mediators, including chemokines and cytokines (4), and these inflammatory mediators mediate the influx of leukocytes into the region of injured myocardium (5). Dysregulated inflammatory responses are known to exert deleterious effects on the heart, including depression of cardiac function (6). However, the impact of aging on myocardial inflammatory responses to I/R is not well characterized, and the mechanism underlying aging-related cardiac dysfunction following I/R remains unclear.

Toll-like receptors (TLRs) are conserved pattern recognition receptors that facilitate the innate immune responses to a variety of pathogens (7, 8). TLRs also recognize endogenous factors released from injured cells, termed as damage-associated molecular patterns (DAMPs) (9). DAMPs have been found to modulate myocardial expression of inflammatory mediators in responses to I/R (10). Excessive inflammatory responses are detrimental since inflammatory mediators are cardiodepressive, activate the pro-apoptotic signaling pathways and cause extracellular matrix destruction and adverse remodeling (11). Inflammatory mediators and leukocytes play important roles in infarct healing and post-ischemic cardiac remodeling (12). Currently, it remains unclear whether TLR2 expression is altered in the aging heart and whether ablation of TLR2 suppresses adverse remodeling and/or improves cardiac functional outcome after myocardial I/R in old subjects.

We hypothesized that TLR2 plays a crucial role in mediating the inflammatory responses and extracellular matrix protein remodeling in aging hearts and that inhibition of TLR2-mediated inflammatory responses improves cardiac function following I/R injury. The purposes of the current study were to determine: (1) the relationship of myocardial inflammatory responses with TLR2 levels in a model of regional myocardial I/R injury in old mice, (2) the effect of TLR2 knockout (KO) on the expression of inflammatory mediators in the myocardium, particularly in the non-ischemic myocardium in aging hearts, (3) the impact of elimination of TLR2-mediated myocardial inflammatory responses on the expression of extracellular matrix proteins in the non-ischemic myocardium of aging hearts, and (4) whether elimination of TLR2-mediated myocardial inflammatory responses in aging hearts reduces myocardial injury and improves cardiac function following I/R.

## Materials and Methods

### Chemicals and Reagents

Antibodies against intercellular adhesion molecule-1 (ICAM-1), vascular cell adhesion molecule (VCAM-1), Glyceraldehyde 3-phosphate dehydrogenase (GAPDH), and β-actin were purchased from Santa Cruz Biotechnology, Inc (Dallas, TX). Antibodies against TLR2, matrix metalloproteinase 9 (MMP 9) and collagen I were purchased from Abcam Inc. (Cambridge, MA). Pam3CSK4 was obtained from InvivoGen (San Diego, CA). Enzyme-linked immunosorbent assay (ELISA) kits for monocyte chemoattractant protein-1 (MCP-1), keratinocyte chemoattractant (KC), and interleukin-6 (IL-6) were purchased from R&D System (Minneapolis, MN). All other chemicals and reagents were purchased from MilliporeSigma (St Louis, MO).

### Animals

Old (20-22 months) and adult (4-6 months) male mice of C57BL/6 and TLR2 KO were acquired from the Jackson Laboratory (Bar Harbor; Maine, USA) and National Institute on Aging (Bethesda, MD, USA), respectively. The TLR2 knockout mice (B6.129-Tlr2^tm1Kir^/J) used in this study are homozygous TLR2 mutant. They grow normally and have no physiological abnormality. For the experiment of testing myocardial TLR2 function, two C57BL/6 adult groups and two C57BL/6 old groups (n=7 per group) were assigned to treatment with normal saline or a specific TLR2 agonist, Pam3CSK4, for 24 hours. For myocardial I/R experiment, three adult groups and three old groups of each C57BL/6 and TLR2 KO mice (n=7 per group) were assigned to one of three treatments: sham surgery, ischemia followed by 3 days of reperfusion and ischemia followed by 14 days of reperfusion. The experiments were approved by the Institutional Animal Care and Use Committee of the University of Colorado Denver [protocol number: B-40516(08)1D], and this investigation conforms to the Guide for the Care and Use of Laboratory Animals (National Research Council, revised 1996).

### Experimental Protocol

Coronary artery ligation was performed to induce regional myocardial I/R. Briefly, mice were anesthetized with a mixture of ketamine (60 mg/kg, ip) and xylazine (12 mg/kg, ip). Anesthetized animals were ventilated with a Harvard rodent respirator *via* tracheal intubation. The body temperature of mice was maintained at 36.8–37.2°C with a thermoregulated surgical board. The chest was opened by a left lateral thoracotomy, and a 7-0 sterile suture was placed around the left descending coronary artery for ligation. The distance from the ligation spot of the left descending coronary artery to the lower edge of the left auricle was about 2.0–3.0 mm. Animals were subjected to 30 min of ischemia, followed by loosening the suture for reperfusion. Then, the chest was closed, and animals were observed for 3 or 14 days. In sham-operated mice, the suture was placed around the left descending coronary artery but untied. Sham-operated animals were observed for the same period of time. Data from sham animals of different time point were combined because sham operation did not cause any significant changes in the analyzed parameters.

In pilot experiments, we examined myocardial ischemia area using Evans blue dye perfusion following coronary ligation. Data from the pilot experiments show that ischemic area is 50.8 ± 6.7% and 46.4 ± 5.9% of left ventricle free wall in young adult mice and old mice, respectively, and there is no significant difference in ischemic area between C57BL/6 and TLR2 KO mice of the same age. Thus, this protocol creates comparable areas of myocardial ischemia in mice of different age and genotype. We did not examine myocardial ischemia area after prolonged reperfusion, at 14 days after ischemia when myocardial injury was analyzed, because of this observation and the concern that the area with blood supply by the ligated coronary segment might have changed due to the formation of scar tissue and the establishment of collateral circulation over time.

### Pressure-Volume Hemodynamic Analysis

Left ventricle function was performed in the mice that was subjected to 14 days of reperfusion. The measurement was conducted following the protocol described previously (13). Pressure-volume loops were recorded for 20 minutes with the MPVS-400 System and PVAN software (Millar Instruments, Houston, TX). At the end of the measurement, hearts were collected, and myocardium specimens from ischemic and non-ischemic (left ventricle tissue above the occlusion site) zones were processed for further analysis.

### Cytokine Assay

Myocardial tissue was homogenized as previously described (14). Levels of MCP-1, KC and IL-6 in ischemic and non-ischemic myocardium were analyzed using enzyme-linked immunosorbent assay (ELISA) kits (R & D Systems, Minneapolis, MN) following the manufacturer’s protocol.

### Immunoblotting

Immunoblotting was performed to analyze the expression of adhesion molecules, MMP 9 and collagen I in non-ischemic myocardium. Myocardial homogenate was fractioned on 4–20% gradient acrylamide gels and transferred onto a nitrocellulose membrane (Bio-Rad Laboratories, Hercules, CA). The membranes were then exposed on X-ray films. GAPDH and β-actin was used for loading control. The area and density of protein bands were analyzed with NIH Image J software.

### Histology

Myocardial tissue was embedded in Tissue-Tek* CRYO-OCT compound and cryosectioned. Transverse tissue sections (5 μm in thickness) were stained with hematoxylin and eosin (H&E) for histological analysis of infarct size. H&E-stained sections were viewed by a blinded viewer. Myocyte-silent space in left ventricle walls were identified as infarct area. Infarct area was calculated as a percentage of total left ventricular wall area. Sections were photographed using a digital camera (Nikon Digital Sight DS-Fi1) mounted on a microscope (Nikon Eclipse 55i). Manual planimetry was performed using the images with Imaging software Nis-Elements Basic Research 4.13 (Nikon) to calculate infarct size.

### Statistical Analysis

StatView software (Abacus Concepts, Calabasas, CA) was used for statistical analysis. Data were expressed as mean ± standard error (SEM). Comparison between two groups was analyzed by Student’s t-test, and difference was confirmed by Nonparametric Mann-Whitney U test. ANOVA with *post hoc* Bonferroni/Dunn test was performed to analyze differences in multiple group comparison, and difference was confirmed by nonparametric Kruskal-Wallis test. A difference was considered significant if *P* value < 0.05.

## Results

### Exacerbated Myocardial Inflammatory Responses to I/R in Old Mice Are Associated With Elevated TLR2 Levels in the Myocardium

We used a moderate myocardial I/R protocol, ligation of LAD at about 0.2 cm below the left auricle for 30 minutes, which causes moderate myocardial injury, but does not result in mortality in young adult mice of either genotype. While we observed approximately 12% mortality in old wild-type mice subjected to this I/R protocol, all old TLR2 KO mice survived after I/R. As the mortality rate is low and the sample size is relatively small, we did not use mortality as an outcome parameter in this study.

**Figure 1:**
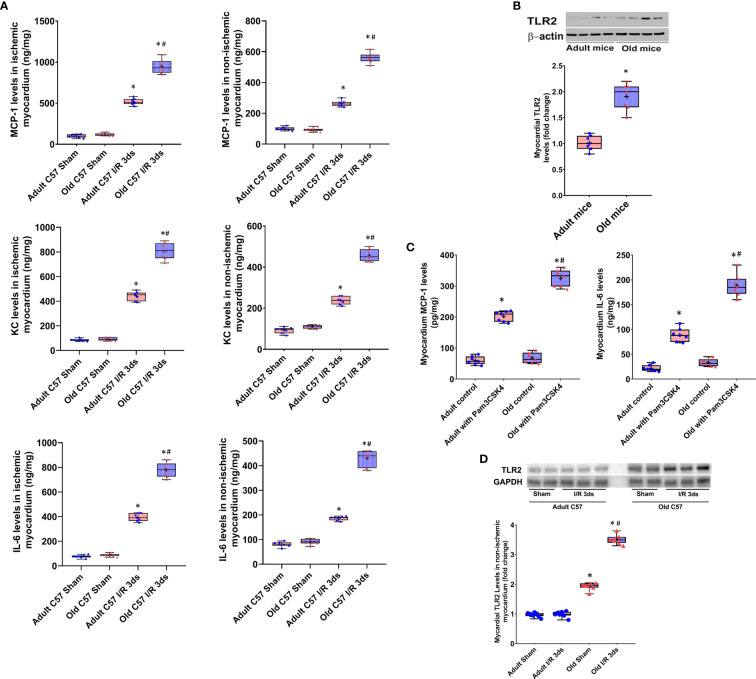
Myocardial hyper-inflammatory responses in old mice are associated with elevated Toll-like receptor (TLR) 2 levels in the heart. **(A)** Levels of monocyte chemoattractant protein 1 (MCP-1), keratinocyte chemoattractant (KC) and interleukin-6 (IL-6) in ischemic (left column) and non-ischemic (right column) myocardium were analyzed at day 3 of reperfusion after 30 minutes of myocardial ischemia induced by left anterior descending artery occlusion. While levels of these inflammatory mediators were increased in the myocardium of both adult and old mice, old mice displayed greater increases in both ischemic and non-ischemic myocardium. N = 7 in each group, *P < 0.05 vs. sham controls, ^#^ *P* < 0.05 vs. adult ischemia/reperfusion. **(B)** Immunoblotting and densitometric analysis revealed higher levels of TLR2 in the myocardium of old mice compared to adult mice. N = 7; *P < 0.05 *vs.* adult. **(C)** Adult mice and old mice were treated with specific TLR2 agonist Pam3CSK4 (2.5 μg/g, iv). Old mice had greater levels of MCP-1 and IL-6 in the myocardium examined at 24 hours after treatment. N = 7; *P < 0.05 vs. saline control; ^#^ *P* < 0.05 vs. adult treated with Pam3CSK4. **(D)** Immunoblotting and densitometric analysis show that TLR2 levels in the non-ischemic myocardium of old mice at day 3 after I/R are greater than control (sham) levels while they are comparable to controls (sham) in adult mice at day 3 after I/R. N = 7; *P < 0.05 *vs.* adult with the same treatment. ^#^ *P *< 0.05 vs. old sham. In **(A–D)**, data are presented as box-and-whiskers plots. The upper and lower borders of the box represent the upper and lower quartiles. The upper and lower whiskers represent the maximum and minimum values of nonoutliers. The horizontal line in the box represents the median, and the + sign represents the mean.

Levels of MCP-1, KC and IL-6 increased markedly in the ischemic and non-ischemic myocardium of wild type mice at day 3 after I/R (**Figure 1A**). Interestingly, levels of these pro-inflammatory cytokines were greater in both ischemic and non-ischemic myocardium of old wild type mice (**Figure 1A**).

To determine the relationship of myocardial hyper-inflammatory responses with cardiac TLR2 levels in old mice, we analyzed TLR2 protein level in the left ventricle myocardium using immunoblotting. **Figure 1B** shows that TLR2 protein levels are higher in the myocardial tissue of old mice in comparison to those of adult mice. To confirm that elevated levels of TLR2 in the hearts of old mice result in a greater myocardial TLR2 responses, we treated adult and old mice with a specific TLR2 agonist Pam3CSK4 (2.5 μg/g, iv) and assessed the production of pro-inflammatory cytokines. The results in **Figure 1C** show that the myocardium of old mice produces higher levels of MCP-1 and IL-6 in responses to TLR2 stimulation. Thus, the elevated levels of TLR2 in aging hearts resulted in greater inflammatory responses to TLR2 stimulation, and the hyper-inflammatory responses of aging hearts to I/R are associated with elevated myocardial levels of functional TLR2.

To understand whether I/R has an impact on myocardial TLR2 levels, we analyzed TLR2 protein levels in non-ischemic left ventricle myocardium collected at day 3 after ischemia and compared them with those in the left ventricle myocardium of sham-treated hearts. Immunoblotting and densitometric analysis confirmed that old mice with sham treatment have higher TLR2 levels in the left ventricle myocardium in comparison to adult sham-treated hearts (**Figure 1D**). Interestingly, TLR2 levels in the non-ischemic myocardium of old mice at day 3 after I/R are greater than control (sham) levels while they are comparable to controls (sham) in adult mice at day 3 after I/R. This finding supports the mechanistic role of elevated expression of TLR2 in the aging heart in mediating the augmented myocardial inflammatory responses to I/R injury.

### TLR2 KO Attenuates the Inflammatory Responses in Both Ischemic and Non-Ischemic Myocardium

**Figure 2:**
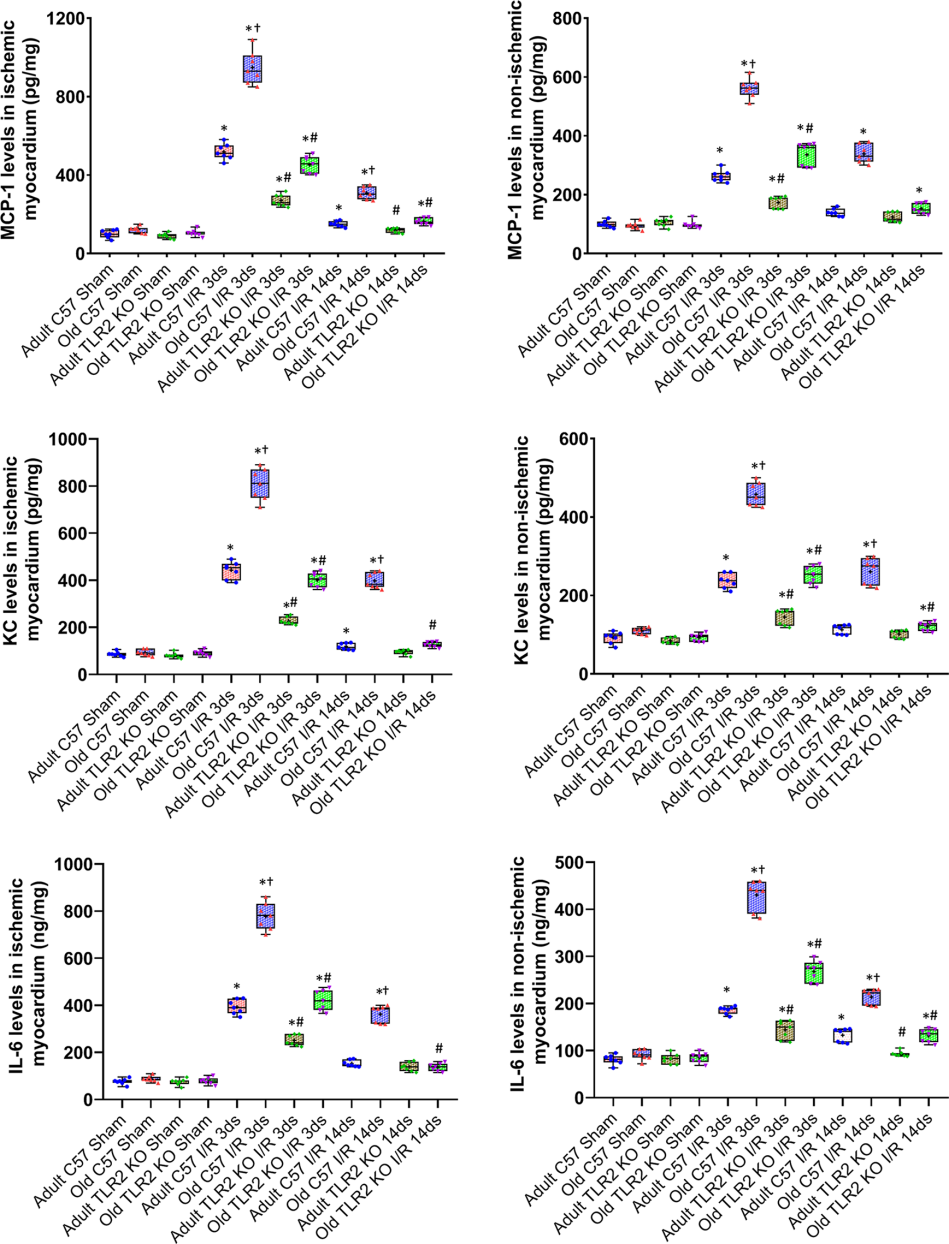
Toll-like receptor (TLR) 2 knockout (KO) has a greater impact on myocardial expression of cytokines in old mice. Myocardial levels of monocyte chemoattractant protein (MCP)-1, keratinocyte chemoattractant (KC), and interleukin (IL)-6 were analyzed at day 3 and 14 of reperfusion after myocardial ischemia with 30 minutes of left anterior descending artery occlusion. TLR2 KO reduced cytokine levels in both ischemic (left column) and non-ischemic (right column) myocardium of adult and old mice, but with a greater reduction in old mice. Data are presented as box-and-whiskers plots; the upper and lower borders of the box represent the upper and lower quartiles; the upper and lower whiskers represent the maximum and minimum values of non-outliers; the horizontal line inside the box represents the median, and the + sign represents the mean. N = 7 in each group, *P < 0.05 vs. combined sham controls (3 and 14 days after ischemia), ^†^ *P < *0.05 vs. C57BL/6 wild type adult mice, *^#^P < *0.05 vs. the same age of wild type mice at the same time point.

**Figure 3:**
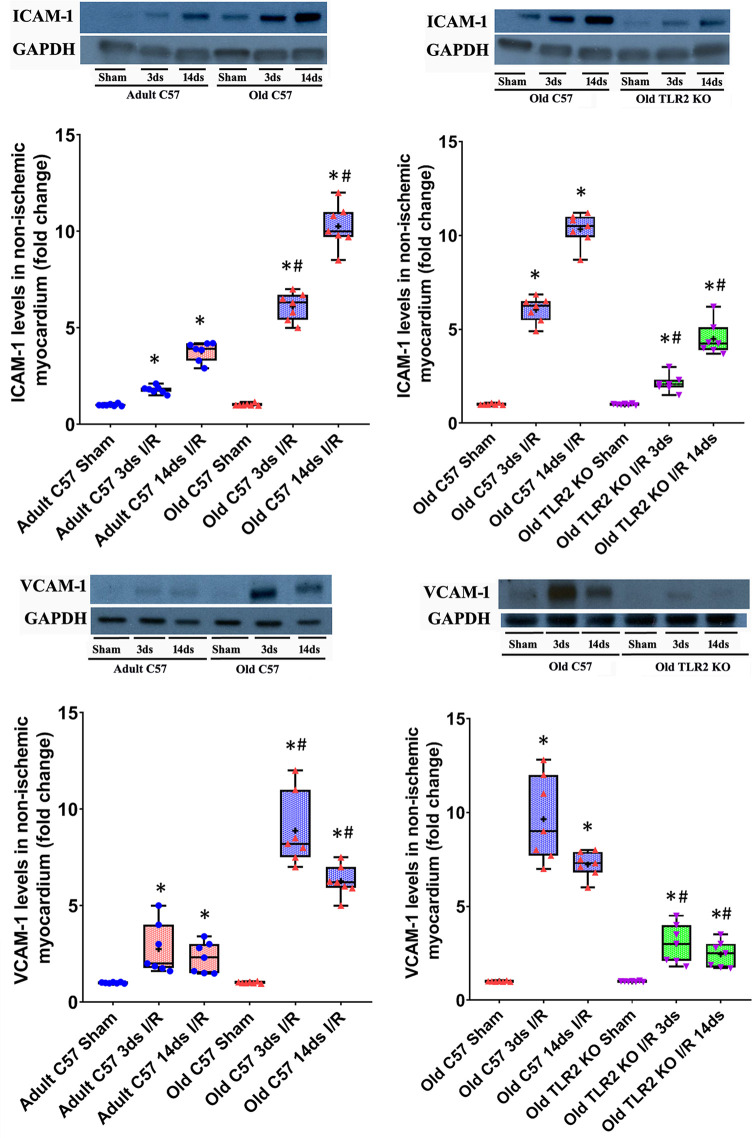
Toll-like receptor (TLR) 2 knockout (KO) suppresses the exaggerated expression of intercellular adhesion molecule (ICAM)-1 and vascular cell adhesion molecule (VCAM)-1 in hearts of old mice. Representative immunoblots and densitometry data show that ICAM-1 and VCAM-1 levels are markedly higher in the non-ischemic myocardium of old C57BL/6 wild-type mice at day 3 and 14 of reperfusion after myocardial ischemia with 30 minutes of left anterior descending artery occlusion in comparison to adult wild type mice. TLR2 KO reduced the levels of these two adhesion molecules in the non-ischemic myocardium of old mice. Data are presented as box-and-whiskers plots where the upper and lower borders of the box represent the upper and lower quartiles; the horizontal line inside the box represents the median; the upper and lower whiskers represent the maximum and minimum values of nonoutliers; and the + sign represents the mean. N = 7 in each group, *P < 0.05 vs. respective sham. ^#^ *P* < 0.05 vs. adult wild type ischemia/reperfusion (I/R) or old wild type I/R.

Myocardial levels of MCP-1, KC, and IL-6 were higher at day 3 and 14 following I/R in both ischemic and non-ischemic myocardium of old wild type mice although the levels of these cytokines were relatively lower in the non-ischemic myocardium (**Figure 2**). TLR2 KO significantly reduced the levels of these inflammatory mediators in the myocardium of old mice. At day 3 of reperfusion, TLR2 KO reduced 41% of MCP-1, 46% of KC and 39% of IL-6 in non-ischemic myocardium in old mice. ICAM-1 and VCAM-1 in the non-ischemic myocardium were also increased at day 3 and 14 in all I/R groups, and the levels were greater in old wild type mice compared to adult wild type mice at the same time point (**Figure 3**). Old TLR2 KO mice had approximately 66% less of ICAM-1 at day 3, and 60% less at day 14 following I/R in the non-ischemic myocardium in comparison to old wild type mice. VCAM-1 levels in the non-ischemic myocardium of TLR KO mice were also lower compared to old wild type mice.

### Old TLR2 KO Mice Display Attenuated Expression of MMP 9 and Collagen I in the Non-Ischemic Myocardium

**Figure 4:**
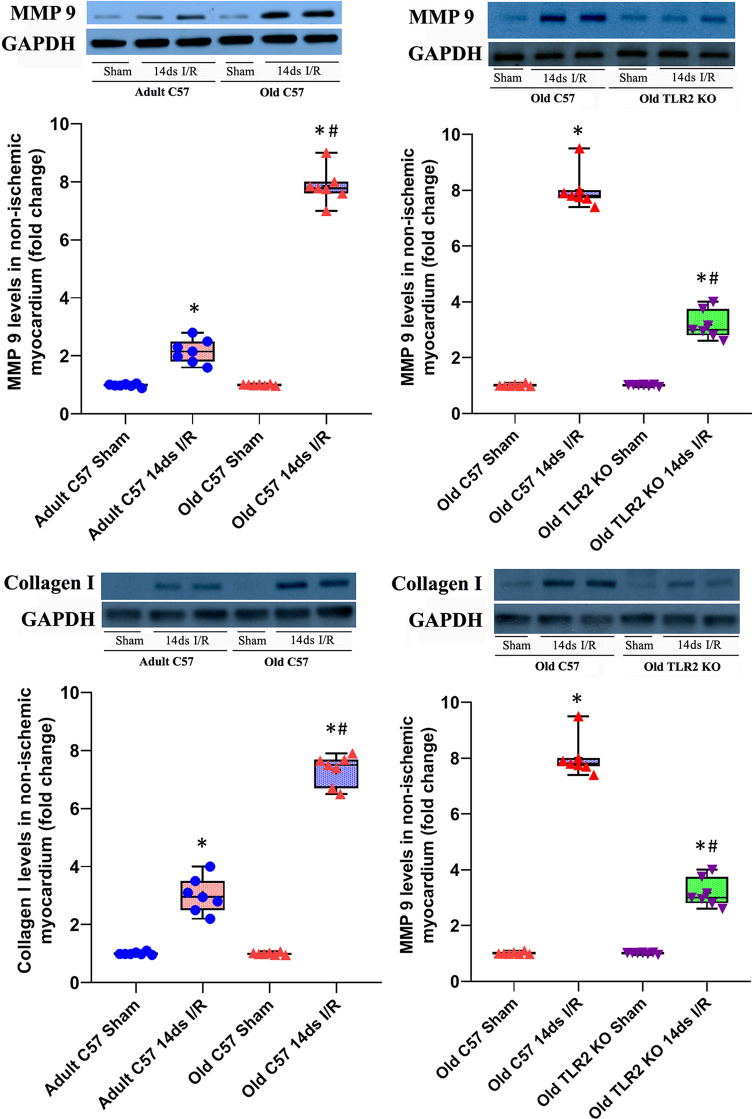
Toll-like receptor (TLR) 2 knockout (KO) attenuates matrix metalloproteinase (MMP) 9 and collagen I expression in old mice. MMP 9 and collagen I levels in the non-ischemic myocardium were analyzed at day 14 of reperfusion after myocardial ischemia with 30 minutes of left anterior descending artery occlusion. Representative immunoblots and densitometry data show greater levels of MMP 9 and collagen I in the non-ischemic myocardium of old C57BL/6 wild-type mice in comparison to adult C57BL/6 wild type mice. Old TLR2 KO mice had significantly lower levels of these two extracellular matrix proteins MMP 9 and collagen I in the non-ischemic myocardium compared to old C57BL/6 wild type mice. Data are presented as box-and-whiskers plots; the upper and lower borders of the box represent the upper and lower quartiles; the upper and lower whiskers represent the maximum and minimum values of nonoutliers; the horizontal line in the box represents the median, and the + sign represents the mean. N = 7 in each group, *P < 0.05 vs. respective sham. ^#^ *P* < 0.05 vs. adult wild-type ischemia/reperfusion (I/R) or old wild type I/R.

We analyzed protein levels of MMP 9 and Collagen I in non-ischemic myocardium in wild-type adult, old, and TLR2 KO old mice at day 14 after I/R (**Figure 4**). MMP 9 protein levels were significantly increased in the non-ischemic myocardium of adult and old wild type mice, and myocardial MMP 9 levels in old wild type mice at day 14 were 2-fold higher than those in adult wild type mice at the same timepoint. TLR2 KO significantly reduced MMP 9 levels in the non-ischemic myocardium of old mice. Collagen I levels in the non-ischemic myocardium had a similar change following I/R. Old wild type mice had much higher collagen I levels at day 14 following I/R compared to adult wild type mice. TLR2 KO markedly suppressed collagen I expression in the non-ischemic myocardium of old mice.

### TLR2 KO Mice Have Smaller Infarct Size

**Figure 5:**
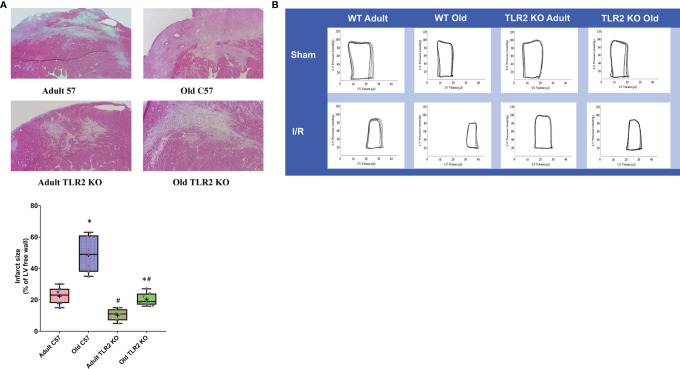
Toll-like receptor (TLR) 2 knockout (KO) reduces infarct size and improves cardiac function in old mice following myocardial ischemia reperfusion (I/R). **(A)** Left ventricle free wall and myocardial infarct area were assessed under a microscope on tissue sections stained with hematoxylin and eosin. Infarct area was identified as the area with myocytes missing. Infarct size is expressed as a percentage of left ventricle free wall. Representative images and quantitative data show that TLR2 KO reduced infarct size in both adult and old mice at day 14 after I/R, but with a greater reduction in old mice. Data are presented as box-and-whiskers plots; the upper and lower borders of the box represent the upper and lower quartiles; the upper and lower whiskers represent the maximum and minimum values of nonoutliers; the horizontal line in the box represents the median, and the + sign represents the mean. N = 7 in each group, ^*^ *P* < 0.05 vs. adult mice of the same strain. ^#^ *P* < 0.05 vs. C57BL/6 wile-type mice of the same age. **(B)** left ventricular function was analyzed by a microcatheter at day 14 after I/R. Representative pressure-volume loops show that old TLR2 KO mice have markedly improved left ventricle functional performance compared to old wild type mice.

HE images of heart tissue sections at day 14 after I/R are shown in **Figure 5A**. Old wild type mice had bigger infarct sizes in comparison to adult wild type mice (56 ± 8% of left ventricle free wall vs. 20 ± 4% of left ventricle free wall, *P*<0.05) at day 14 after I/R. TLR2 KO reduced the infarct size in old mice (21 ± 3% in old TLR2 KO mice vs. 56 ± 8% in old wild type mice, *P*<0.05) and in adult mice (11 ± 2% in adult TLR2 KO mice vs. 20 ± 4% in adult wild type mice). However, the reduction was greater in old TLR2 KO mice (63%) than in adult TLR2 KO mice (45%).

### Old TLR2 KO Mice Display Improved Left Ventricle Function

We analyzed left ventricle performance at day 14 after I/R using a microcatheter. The pressure-volume loops presented in **Figure 5B** show a right shift in I/R groups compared to their sham controls due to increased end-systolic and end-diastolic volume. However, TLR2 KO I/R groups, especially the old mice, had greater loop areas than the corresponding wild type I/R groups, indicating reduced myocardial injury and attenuated adverse remodeling.

The left ventricle function parameters are summarized in **Table 1**. There was no significant difference in left ventricle function among sham groups, including adult wild type, old wild type, adult TLR2 KO and old TLR2 KO groups. Compared to wild type sham groups, both adult and old wild type I/R groups had decreased developed pressure, ejection fraction, and cardiac output at day 14 following I/R. But old wild type mice had poorer left ventricle function parameters at day 14 compared to adult wild type mice. Old TLR2 KO mice showed significantly improved left ventricle function compared to old wild type mice in terms of developed pressure (68 ± 3 vs. 58 ± 5 mmHg; *P*<0.05), ejection fraction (32.1 ± 2.1 vs. 24.4± 2.0%; *P*<0.05), and cardiac output (5.7 ± 0.4 vs. 4.6 ± 0.2 ml/min; *P*<0.05). Furthermore, adult TLR2 KO mice had improved left ventricle performance at day 14 in comparison to adult wild type mice. However, TLR2 KO had a greater benefit in old mice than in adult mice.

**Table 1:** Left ventricle function parameters at day 14 after ischemia/reperfusion (I/R).

	Sham				I/R			
	Wild Type Adult	Wild Type Old	TLR2 KO Adult	TLR2 KO Old	Wild Type Adult	Wild Type Old	TLR2 KO Adult	TLR2 KO Old
Heart Rates (bpm)	480 ± 30	478 ± 36	485 ± 44	489 ± 35	479 ± 25	481 ± 31	485 ± 44	473 ± 22
Developed Pressure (mmHg)	85 ± 6	83 ± 7	87 ± 5	82± 6	69 ±4*	58 ± 5*†	79 ± 4#	68 ± 3*#
End-systolic Volume (µL)	7.9 ± 0.8	8.4 ± 0.6	7.7 ± 0.5	7.6 ± 0.6	21.0 ± 3*	31.2 ± l.8*†	17.3 ± 1.2*#	25.5 ± 1.8*#
End-diastolic Volume (µL)	22.0 ± 2.0	21.8 ± 2.0	21.2 ± 1.8	20.8 ± 1.8	32.3 ± 2.1*	41.1 ± 2.0*†	29.8 ± 2.1*	36.1 ± 1.3*#
Ejection Fraction (%)	64.1 ± 5.8	61.5 ± 6.3	63.7 ± 4.2	63.5 ± 4.8	35.0 ± 1.5*	24.4 ± 2.0*†	42.9 ± 3.7*#	32.1 ± 2.1*#
Cardiac Output (mL/min)	6.8 ± 0.7	6.5 ± 0.5	6.6 ± 0.5	6.5 ± 0.4	5.4 ± 0.2*	4.6 ± 0.2*†	6.0 ± 0.3#	5.7 ± 0.4*#

Mean ± SE; n=7 in each group. ^*^P< 0.05 vs. respective sham; ^†^P< 0.05 vs. C57BL/6 wild type adult; ^#^P< 0.05 vs. wild type of the same age.

## Discussion

In this study, we found that the remote non-ischemic myocardium of aging hearts expresses greater levels of cytokines and adhesion molecules at day 3 and 14 following regional I/R, which correlates with elevated TLR2 levels in the aging hearts. The exacerbated inflammatory responses in the non-ischemic myocardium of aging hearts are associated with over-expression of extracellular matrix proteins, as well as bigger infarct size and worse left ventricle dysfunction at day 14 following I/R. Hearts of old TLR2 KO mice display markedly attenuated inflammatory responses and extracellular matrix protein remodeling in the remote non-ischemic myocardium and have smaller infarct size and significantly improved left ventricle function. These results demonstrate that elevated TLR2 levels in aging hearts play a mechanistic role in the exacerbated myocardial inflammatory responses, exaggerated myocardial injury and left ventricle dysfunction following regional I/R.

Timely reperfusion is a strategy to prevent progression of myocardial necrosis. However, reperfusion also paradoxically induces additional injury. Reperfusion injury could be at least partly attributed to tissue inflammatory responses induced by reperfusion (15). Particularly, myocardial I/R evokes the inflammatory responses that include complement activation, cytokine production and leukocyte infiltration (13, 16). However, few studies have investigated the inflammatory changes in aging hearts after myocardial I/R injury. MCP-1 and IL-8 (KC in murine) are the key chemokines that regulate leukocyte migration and infiltration (17, 18). ICAM-1 and VCAM-1 are critical molecules for leukocyte adhesion (19). In this study, we found that MCP-1, KC, IL-6, and adhesion molecule levels are markedly increased at day 3 and 14 of reperfusion in the remote non-ischemic myocardial tissue in hearts of adult and old wild type mice. Interestingly, the levels of these inflammatory mediators are much higher in old wild type mice in comparison to those in adult wild type mice. These results indicate that the remote non-ischemic myocardium has long-lasting inflammatory responses to I/R insult and that aging hearts have greater inflammatory responses to I/R injury. The exacerbated myocardial inflammatory responses in aging hearts lead to greater injury and worse left ventricle dysfunction.

Although exaggerated inflammatory responses have been found in elderly people in several studies (20–22), the mechanism underlying age-related hyper-inflammatory responses remains unclear. TLR2 is expressed in cardiomyocytes (23) and endothelial cells (24). Upon ligand binding (either pathogen-associated molecular patterns or damage-associated molecular patterns), TLR2 induces NF-κB activation, resulting in the production of inflammatory mediators (25). Interestingly, we observed that TLR2 protein levels are elevated in the hearts of old wild type mice, and the hearts of old wild type mice produce greater levels of inflammatory cytokines in response to a specific TLR2 agonist. Thus, aging hearts have higher levels of functional TLR2. Our findings are consistent with a report that shows increased levels of TLR2 in kidneys of aging rats (26). Furthermore, we observed that TLR2 levels in non-ischemic left ventricle myocardium of old mice at day 3 after I/R are greater in comparison to those in left ventricle myocardium of aging hearts subjected to sham treatment. The increase in myocardial TLR2 levels correlates with greater inflammatory responses in the non-ischemic myocardium of aging hearts following I/R injury.

It appears that elevated levels of TLR2 in aging hearts exacerbate the inflammatory responses to I/R injury. Indeed, our results show that TLR2 KO has a bigger effects on myocardial production of inflammatory cytokines and adhesion molecules in aging hearts, resulting in significantly lower levels of MCP-1, KC, IL-6, ICAM-1 and VCAM-1 in the non-ischemic myocardium at day 3 and 14 following I/R. This finding demonstrates that activation of myocardial TLR2 contributes to the mechanism underlying myocardial inflammatory responses, and higher levels of myocardial TLR2 contribute to the exacerbated myocardial inflammatory responses in aging hearts. However, it remains unclear from this study how aging and I/R up-regulate myocardial TLR2 expression. Inflammatory mediators may enhance TLR2 expression and thereby augment myocardial inflammatory responses to I/R injury. Future studies are needed to elucidate the underlying mechanisms.

Even though the levels of inflammatory mediators remain higher, especially in aging hearts, at day 14 after I/R, MCP-1, KC and IL-6 levels declined from the levels at day 3. However, it is noteworthy that the levels of ICAM-1 increased from day 3 to day 14. This not only shows sustained inflammatory responses in the non-ischemic myocardium, but also indicates a role of ICAM-1 in cardiac remodeling as myocardial fibroblasts, collagen deposition and scar maturation are the dominant events at day 14 following I/R (27). It is known that ICAM-1 also functions as a receptor for the integrins on the surfaces of leukocytes and transduces signals for cell activation (28). The interaction of leukocytes with fibroblasts and myofibroblasts through the integrin/ICAM-1-dependent mechanism may promote extracellular matrix remodeling and myocardial fibrosis.

MMPs and collagens are the components of extracellular matrix in myocardium, and MMPs have proteolytic functions (29). During the early stages of myocardial I/R injury, degradation of extracellular matrix proteins by MMPs results in infarct expansion, ventricular wall thinning and ventricular dilation (30). Excess collagen type I synthesis and deposition contributes to the enhancement of myocardial fibrosis and deterioration of cardiac function (31). In the present study, we found that levels of MMP 9 and collagen I are increased in the non-ischemic myocardium at day 14 following I/R, especially higher in old wild type mice. Interestingly, MMP 9 and collagen I levels was markedly reduced in old TLR2 KO mice at day 14 after myocardial I/R. Importantly, we observed in this old animal I/R model that lower protein levels of MMP 9 and collagen I in the non-ischemic myocardium in TLR2 KO old mice correlate to improved left ventricle functional performance. Apparently, TLR2-mediated myocardial inflammatory responses in the non-ischemia areas and associated adverse extracellular matrix remodeling contributed to the worse dysfunction of aging hearts. It should be noted that old wild type mice have larger infarct sizes at day 14 after I/R, and that TLR2 KO reduced infarct size in old mice. Therefore, reduced myocardial I/R injury and suppressed extracellular matrix remodeling in the remote non-ischemic myocardium should be responsible for the improvement of cardiac function in old TLR2 KO mice. Alternately, both augmented injury and adverse remodeling induced by TLR2-mediated myocardial inflammatory responses in aging hearts exacerbate cardiac dysfunction.

Exaggerated myocardial inflammatory responses are detrimental in the setting of myocardial ischemic injury. Enhanced myocardial inflammatory responses have been observed in elderly patients undergoing cardiac surgery (32) Tissue damage, endotoxemia, contact of blood with an artificial surface associated with cardiopulmonary bypass may trigger myocardial inflammatory responses in patients undergoing open-heart surgery (33). The responses are exacerbated in the elderly, and inflammatory mediators have greater detrimental effects on hearts of elderly patients (34). A retrospective study showed that increased inflammatory responses in aging during the postoperative period after coronary artery bypass surgery caused 4 times higher post-surgery mortality in elderly patients (35). The findings of the present study suggest an important mechanistic role of TLR2 in mediating myocardial inflammatory responses to ischemic injury, as well as up-regulation of myocardial TLR2 by aging. It appears that inhibition of the activity of TLR2 signaling pathway improves the cardiac functional outcome after myocardial ischemia. Currently, multiple approaches, including TLR2 blocking antibodies, specific siRNAs, peptide inhibitors are capable of inhibiting TLR2 signaling to reduce the systemic and myocardial inflammatory responses mediated by TLR2. These agents may have therapeutic potential for improving the clinical outcome in elderly patients sustaining myocardial I/R injury. Future studies are needed to evaluate the effect of specific TLR2 blockade in reducing the inflammatory responses and improving the cardiac function in the elderly following myocardial I/R injury.

It should be noted that this study has several limitations. Understanding of aging-associated alterations in myocardial inflammatory responses to ischemic injury, injury repair and cardiac remodeling is critical for designing precise strategies to protect against myocardial injury and promote myocardial repair in elder patients with acute myocardial ischemic attack or undergoing cardiac surgery with obligatory myocardial ischemia. However, the age-related changes in myocardial inflammatory responses in mice may not exactly correlate with those in humans. Furthermore, the heart function data obtained in this study reflect global left ventricular function, not the regional function in the area of injury. Further, other factors not addressed in this study may contribute to injury and dysfunction in aging hearts, and TLR2 KO may suppress the production of those factors.

## Conclusion

Elevated levels of myocardial TLR2 exaggerate the inflammatory responses after I/R injury in old wild type mice and result in bigger infarct size and worse cardiac dysfunction. MCP-1, KC, IL-6, ICAM-1 and VCAM-1 are markedly increased in the non-ischemic myocardium of old wild type mice at day 14 following I/R, which is associated with excessive expression of MMP 9 and collagen I. TLR2 KO attenuates the inflammatory responses in both ischemic and non-ischemic myocardium, suppresses altered extracellular matrix protein expression in non-ischemic myocardium, reduces myocardial injury and improves left ventricle function in aging hearts following myocardial I/R. Pharmacological down-regulation of TLR2 activity may suppress aging-related myocardial hyper-inflammatory responses to improve cardiac outcome in the elderly inflicted by acute myocardial I/R injury.

## Data Availability Statement

The original contributions presented in the study are included in the article/supplementary material. Further inquiries can be directed to the corresponding author.

## Ethics Statement

The animal study was reviewed and approved by Institutional Animal Care and Use Committee of the University of Colorado Denver.

## Author Contributions

Conceptualization, XM. Methodology, YZ and LA. Investigation, YZ, LA, QY, and ET. Data Acquisition and Analysis, YZ, LA, QY, and ET. Writing - Original Draft Preparation, YZ. Writing - Review and revision, XM and DF. Supervision, XM and DF. Project Administration, XM. Funding Acquisition, XM. All authors have read and approved the final version of this manuscript.

## Funding

This work was supported in part by the National Institute of Health grant GM129641.

## Conflict of Interest

The authors declare that the research was conducted in the absence of any commercial or financial relationships that could be construed as a potential conflict of interest.

## Publisher’s Note

All claims expressed in this article are solely those of the authors and do not necessarily represent those of their affiliated organizations, or those of the publisher, the editors and the reviewers. Any product that may be evaluated in this article, or claim that may be made by its manufacturer, is not guaranteed or endorsed by the publisher.

## Abbreviations

I/R, ischemia/reperfusion; TLR, Toll-like receptor; KO, knockout; MCP, monocyte chemoattractant protein; KC, keratinocyte chemoattractant; IL, interleukin; ICAM, intercellular adhesion molecule; VCAM, vascular cell adhesion molecule; MMP, matrix metalloproteinase; ELISA, enzyme-linked immunosorbent assay; GAPDH, Glyceraldehyde 3-phosphate dehydrogenase.
